# Recent advances in Del Nido cardioplegia: A comprehensive analysis of randomized clinical trials in adult cardiac surgery

**DOI:** 10.1097/MD.0000000000039453

**Published:** 2024-09-06

**Authors:** Muhammad Ahmed, Adarsh Raja, Vikash Virwani, Sandesh Raja, Syed Yawar Hussain, Abdul Moeed, Shanta Bai, Johar Abbas, Mohammed Mahmmoud Fadelallah Eljack, Muhammad Sohaib Asghar

**Affiliations:** a Department of Internal Medicine, Shaheed Mohtarma Benazir Bhutto Medical College Lyari, Karachi, Pakistan; b Department of Internal Medicine, Aga Khan University and Hospital, Karachi, Pakistan; c Department of Internal Medicine, Dow University of Health Sciences, Karachi, Pakistan; d Department of Internal Medicine, Liaquat National Hospital and Medical College, Karachi, Pakistan; e University of Bakhtalruda Faculty of Medicine and Health Sciences, Al-Dewaym, Sudan; f Division of Nephrology and Hypertension, Mayo Clinic, Rochester, MN.

**Keywords:** adults, cardioplegia, Del Nido, meta-analysis, surgery

## Abstract

**Background:**

Del Nido cardioplegia (DNC) has extensively been used for pediatric population undergoing cardiac surgery. However, its use in adult cardiac surgeries have been limited thus, its benefits are not yet fully known. This analysis was performed to evaluate the impact of DNC versus any other type of cardioplegia in adult patients who are undergoing cardiac surgery.

**Methods:**

We systematically searched PubMed, Cochrane Library, and Scopus from database inception till March 2023, and moderate to high-quality randomized controlled trials were included which compared DNC to other cardioplegia. The primary outcome was postoperative stroke and/or transient ischemic attack (TIA). Secondary outcomes included spontaneous rhythm return, postoperative myocardial infarction, all-cause mortality, postoperative atrial fibrillation, defibrillation after coronary reperfusion, postoperative intra-aortic balloon pump, postoperative kidney injury, postoperative low cardiac output syndrome, inotropic support, cardiopulmonary bypass time, cross-clamp time, blood transfusion, cardioplegia volume, hospital stay, intensive care unit stay, mechanical ventilation stay, postoperative left ventricular ejection fraction, and cardiac markers.

**Results:**

In this meta-analysis, 13 studies were included with a patient population of 2207. Stroke and/or TIA studies (risk ratio [RR]: 0.54, 95% CI [0.29, 1.00]) and all-cause mortality studies (RR: 1.30, 95% CI [0.66, 2.56]) were insignificant. From the secondary outcomes, spontaneous rhythm return (RR: 1.58, 95% CI [1.02, 2.45]), defibrillation after coronary reperfusion (RR: 0.49, 95% CI [0.30, 0.79]), inotropic support (RR: 0.70, 95% CI [0.57, 0.85]), composite risk of stroke and/or TIA and/or acute kidney injury and mortality (RR: 0.72, 95% CI [0.53, 0.99]), cross-clamp time (mean difference [MD]: −6.01, 95% CI [−11.14, −0.89]), blood transfusion (RR: 0.73, 95% CI [0.60, 0.90]), cardioplegia volume (MD: −537.17, 95% CI [−758.89, −315.45]), troponin T (MD: −1.71, 95% CI [−2.11, −1.32]), creatine phosphokinase-MB (MD: −2.96, 95% CI [−5.84, −0.07]) were significant. Whereas all other secondary outcomes were found to be insignificant.

**Conclusion:**

No significant difference was observed between patients undergoing Del Nido administration in comparison to other cardioplegia solutions for the primary outcome, stroke or/and TIA.

## 1. Introduction

Myocardial injury during cardiac surgery is the leading cause of death and morbidity, accounting for 20% to 30% of perioperative mortality and significant complications.^[1]^ The primary objective of myocardial protection in cardiac surgery is to maintain myocyte viability during ischemia and to restore energetic activities upon reperfusion and various means of protection have been developed.^[2,3]^ Cardioplegia solutions are central to myocardial protection, inducing diastolic cardiac arrest, reducing oxygen consumption, and minimizing ischemia-reperfusion injury and without effective protection, patients face higher risks of postoperative dysfunction, arrhythmias, and heart failure, impacting recovery and long-term outcomes.^[4]^

Cardioplegia is theoretically focused on an electrical gradient of ions across the myocardial cell membrane to produce diastolic cardiac arrest and prevent ischemia-reperfusion harm.^[5]^ In cardiac surgeries, a wide range of cardioplegia treatments for myocardial protection are now available. Based on simple classifications, there are 2 primary types of cardioplegia solutions: crystalloid cardioplegia and basic solutions that comprise the constituents of blood.^[6]^

The Del Nido cardioplegia (DNC) is a crystalloid solution (Plasma-Lyte A, lidocaine, magnesium sulfate, potassium chloride, mannitol, sodium bicarbonate) which is mixed with blood in a 1:4 ratio (blood/crystalloid). This cardioplegia provides excellent protection for 90 minutes of cross-clamp duration when administered at a dosage of 20 mL/kg.^[7,8]^ It aids in the scavenging free radicals, preserving high-energy phosphate, providing fast depolarization, and having antiarrhythmic impact.^[9–12]^ Del Nido solution has recently been found to be safe and effective in adult patients following heart surgery.^[13–15]^ However, in adult cardiac surgery, it is uncertain whether DNC has a significant benefit over standard cardioplegia.

Previous meta-analyses focused on evaluating the benefits of DNC compared to other cardioplegia methods.^[16]^ These analyses considered a variety of outcomes. We have improved on this study by including more critical outcomes, such as spontaneous rhythm return, troponin T levels, postoperative intra-aortic balloon pump (IABP) usage, inotropic support, cardioplegia volume, and postoperative troponin I, troponin T, and creatine phosphokinase-MB (CK-MB) levels at different time intervals.

Our study represents an updated systematic review and meta-analysis of all randomized studies comparing Del Nido with other cardioplegic solutions. The aim is to determine the benefits of the Del Nido solution on clinically relevant outcomes that have received little attention.

## 2. Methods

This meta-analysis was conducted by following Preferred Reporting Items for Systematic Review and Meta-analysis guidelines.^[17]^

### 2.1. Data sources and search strategy

A comprehensive literature search was conducted on PubMed, Scopus, and Cochrane Library databases from inception to March 2023. Literature search was conducted without any limitations on age, language, time, or sample size. The following keywords and their MeSH terms were used in the thorough literature search: “Del Nido cardioplegia,” “Cardioplegia solutions,” “Adult cardiac surgery.” Detailed search strategies used in these databases is provided in the Table S1, Supplemental Digital Content (http://links.lww.com/MD/N419).

### 2.2. Study selection

All articles retrieved from the databases were transferred to the Endnote X9 (Clarivate Analytics, US), to remove the duplicated articles. Two individual reviewers (AR and YHS) carefully screened the articles, initially by title and abstract, and afterwards full text articles were examined. In case of any disagreement, a third reviewer (VV) was consulted. Our study adhered to the PICO guidelines, where our population consisted of individuals undergoing various cardiac surgeries, the intervention involved DNC, the control group received different types of cardioplegia, and the outcomes are detailed below. Studies were selected on following inclusion criteria: (a) randomized controlled trials (RCTs) that compared Del Nido versus other cardioplegia solutions in patients who underwent cardiac surgery, (b) patients aged 18 years and above, (c) studies with at least 1 outcome of interest, (d) outcomes of interest were reported which included: stroke and/or transient ischemic attack (TIA), spontaneous return of rhythm, postoperative myocardial infarction (MI), mortality, postoperative atrial fibrillation (POAF), defibrillation after coronary reperfusion, postoperative IABP, postoperative kidney injury, postoperative low cardiac output syndrome (LCOS), inotropic support, composite risk of stroke and/or TIA and/or acute kidney injury and mortality, cardiopulmonary bypass (CPB) time, cross-clamp time, blood transfusion, cardioplegia volume, hospital stay, intensive care unit (ICU) stay, mechanical ventilation time, postoperative left ventricular ejection fraction (LVEF), and cardiac biomarkers (troponin T, troponin I, and CK-MB).

The primary endpoints in our comparison between DNC and other types of cardioplegia is stroke and/or TIA and all the other outcomes are secondary which include myocardial protection (troponin and CK-MB levels), cardiac function (postoperative LVEF, need for inotropic support, and spontaneous return of rhythm), duration of CPB and cross-clamp time, and postoperative complications (such as POAF, defibrillation after coronary reperfusion, postoperative IABP, MI, LCOS, kidney injury, cardioplegia volume, need for transfusion, ventilation time, hospital and ICU stay).

### 2.3. Data extraction and quality assessment

Two reviewers (AR and YHS) conducted the data extraction of selected studies. The following data was extracted from each relevant study: (a) study name and year, (b) mean age of patients in each group, (c) the number of patients in each group (Del Nido vs other cardioplegia), (d) all outcomes of interest. For cardiac markers, the mean values reported in different studies were at different time intervals. In instances where the study did not directly report the mean, the data was inferred through extrapolation from the provided graphs. Cochrane Collaboration’s risk of bias 2.0 (RoB 2.0) tool was used to assess the risk of bias in RCTs.^[18]^

### 2.4. Statistical analysis

For statistical analysis Review Manager (RevMan Version 5.4.1) provided by Cochrane Collaboration Network was used. A random-effects meta-analysis was performed to derive risk ratios (RRs) and their corresponding 95% confidence intervals (CIs). Similarly, a random-effects meta-analysis was conducted for continuous outcomes to obtain mean difference (MD) and their 95% CIs. Meta-regression was performed using Comprehensive Meta Analysis (Version 3.3.070) for the outcome of stroke and/or TIA with the following moderators: female sex % and baseline comorbidities including diabetes mellitus and hypertension. Higgins I^2^ was used to measure heterogeneity and the value of I^2^ = 25% to 50% was considered mild heterogeneity, 50% to 75% was considered moderate and >75% was considered severe heterogeneity.^[19]^ Continuous data provided in median and interquartile range was converted to mean and standard deviation using Wan method.^[20]^ A *P*-value <.05 was considered statistically significant. Funnel plots were constructed for the outcomes having more than 10 studies.^[21]^

## 3. Results

### 3.1. Study selection and characteristics

**Figure 1.**
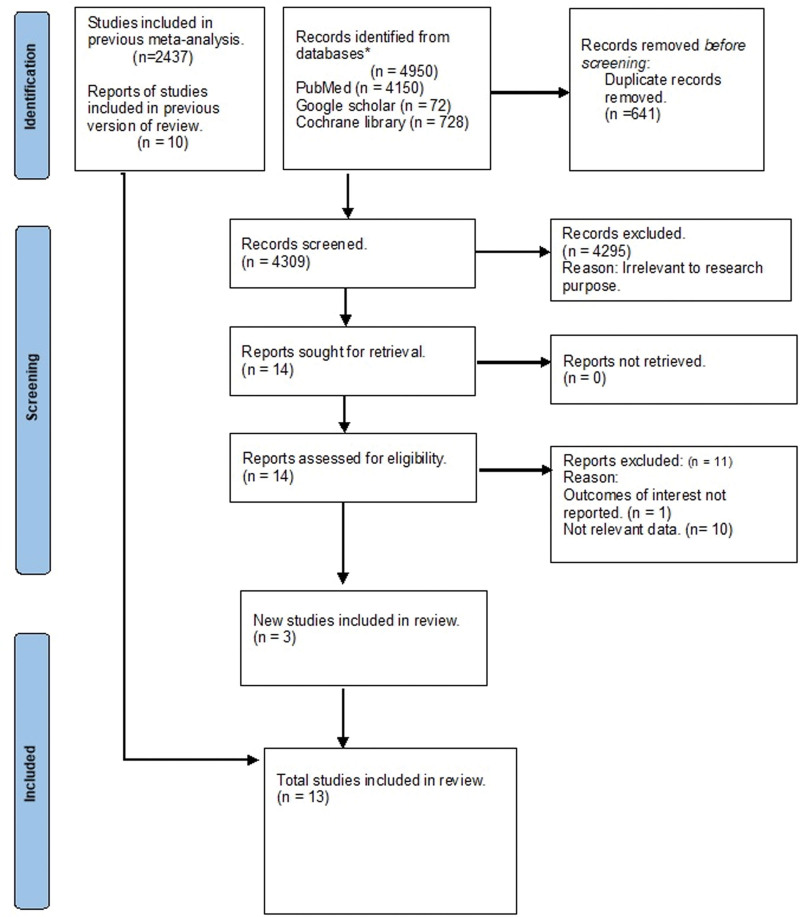
Preferred Reporting Items for Systematic Review and Meta-analysis (PRISMA) flowchart.

The literature search yielded 5241 results. After carefully screening and excluding the duplicate articles, we were left with 13 studies which were included in the final meta-analysis. The results of the literature search are summarized in the form of a Preferred Reporting Items for Systematic Review and Meta-analysis flowchart (Fig. 1). The number of patients from these 13 studies totaled 2207 (1068 Del Nido and 1139 control). The mean age of the patients in Del Nido group was 61.23 years, and 61 years in the control group. The baseline characteristics of the patients included in each study are presented in Table 1. Studies that included both valve and/or coronary artery bypass grafting (CABG) surgeries were by Ad, Demir, Jiang, and Rizvi. Studies focused exclusively on valve surgeries were by Gunyadin and Santera. Studies that included only CABG surgeries were by Ucak, Urcun, Kirişci, and Lama. Finally, studies that involved mixed procedures, including valve, CABG, and other surgeries, were by Garcia-Suarez, Mehrabanian, and Zhang.

**Table 1:** General characteristics of included studies and patients.

Study	Publication year	Journal	Patients Del Nido/Control	Control	Procedure type	Timing of Cardioplegia	Route of Cardioplegia	Temperature Del Nido °C	Temperature Control °C
Ad et al^[12]^	2017	J Thorac Cardiovasc Surg	48/41	Whole blood	Valve/CABG	Intermittent	Unknown	6–10	8–10
Garcia-Suarez et al	2022	J Thorac Cardiovasc Surg	232/239	Whole blood	Valve/CABG/Aortic/Myectomy	Intermittent	Antegrade/retrograde	4	4
Gunyadin et al	2020	Innovations (Phila)	95/96	Custodiol	Valve	Intermittent	Unknown	4–8	4–8
Demir et al^[22]^	2022	Niger J Clin Pract	106/107	Crystalloid with blood	Valve/CABG	Unknown	Unknown	4–10	4–10
Mehrabanian et al	2018	Iran Red Crescent Med J	21/19	Custodiol	CABG/AVR/MVR	Unknown	Antegrade	Unknown	Unknown
Santera et al	2020	J Thorac Cardiovasc Surg	75/75	Cold blood	Valve	Unknown	Unknown	Unknown	Unknown
Ucak et al	2018	Ann Thorac Cardiovasc Surg	112/185	Warm blood	CABG	Intermittent	Antegrade/retrograde	4	33–34
Urcun et al	2021	Heart Surg Forum	150/150	Whole blood	CABG	Intermittent	Antegrade/retrograde	4	Unknown
Jiang et al	2019	Zhonghua Yi Xue Za Zhi	44/40	St. Thomas’ hospital	Valve/CABG	Unknown	Unknown	Unknown	Unknown
Rizvi et al	2022	Pak J Med Sci	40/40	St. Thomas’ hospital	Valve/CABG	Intermittent	Unknown	4	4
Kirişci et al	2020	Turk J Med Sci	30/30	Cold blood	CABG	Intermittent	Antegrade	4	4
Moktan Lama et al	2021	Perfusion	45/45	St. Thomas’ hospital	CABG	Unknown	Antegrade/retrograde	4–6	4–6
Zhang et al	2022	Ann Thorac Cardiovasc Surg	65/68	St. Thomas’ hospital	CABG/AVR/MVR/MVP	Unknown	Antegrade	4–6	10–12

AVR = aortic valve replacement, CABG = coronary artery bypass graft, MVR = mitral valve replacement, MVP = mitral valvuloplasty.

### 3.2. Quality assessment

Quality assessment for the included RCTs was done using Cochrane Risk of Bias tool (RoB 2.0). All the included studies had low to moderate risk of bias. Detailed quality assessment is provided in the Figure S1, Supplemental Digital Content (http://links.lww.com/MD/N419).

### 3.3. Primary outcome

#### 3.3.1. Stroke and/or TIA

**Figure 2.**
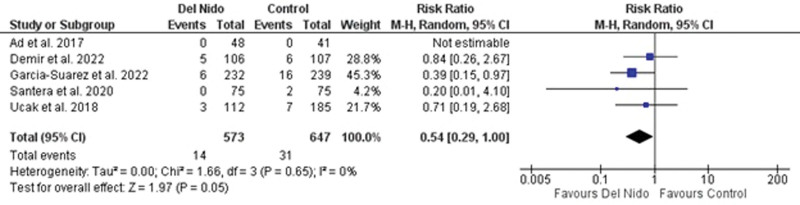
Forest plot of stroke and/or TIA.

Six of the 13 included RCTs reported the incidence of stroke and/or TIA and were included in the primary outcome analysis. No significant difference was observed between Del Nido and control group (RR: 0.54, 95% CI [0.29, 1.00]; *P* = .05; I^2^ = 0%) (Fig. 2).

### 3.4. Secondary outcomes

**Figure 3.**
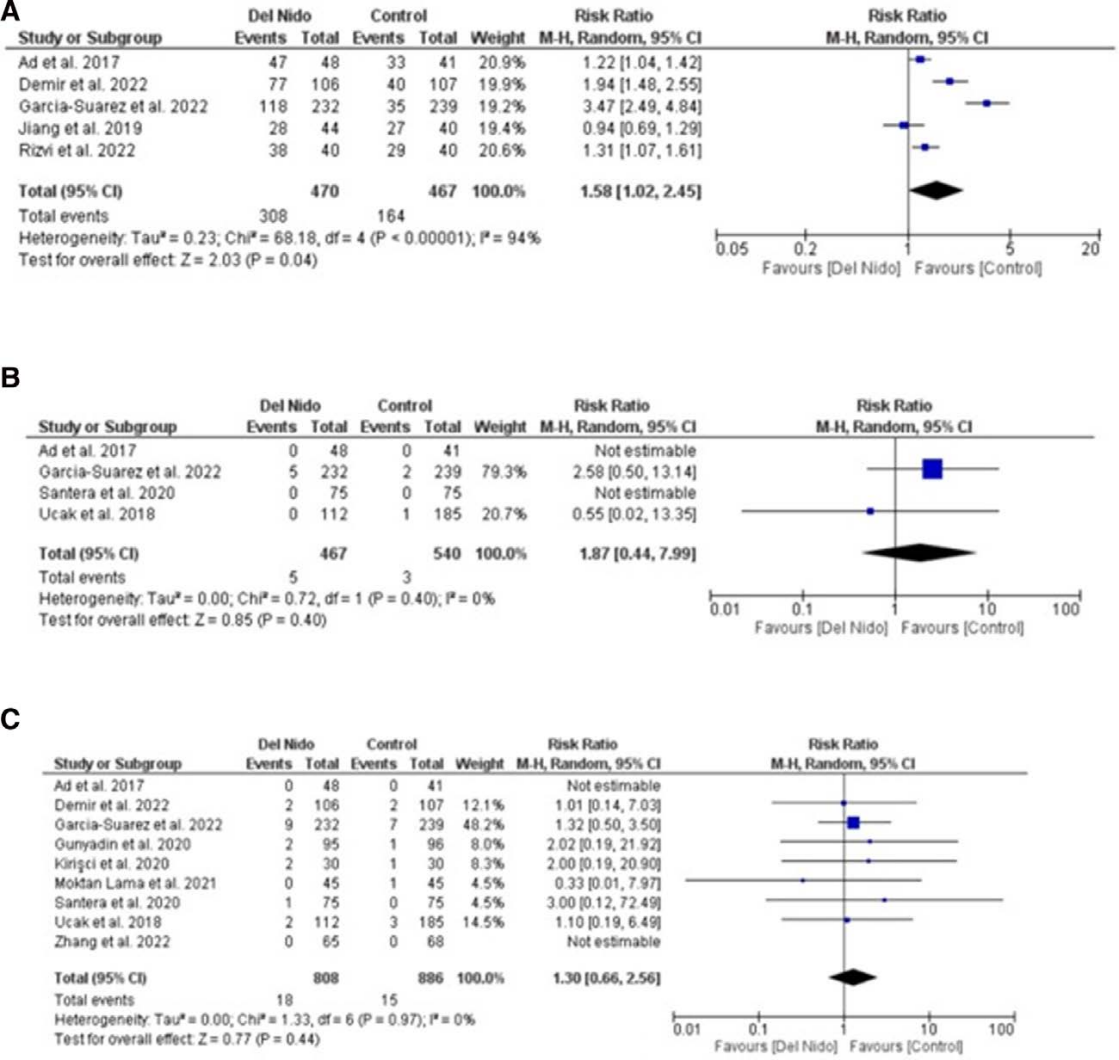
(A) Spontaneous rhythm return, (B) myocardial infarction, and (C) mortality.

**Figure 4.**
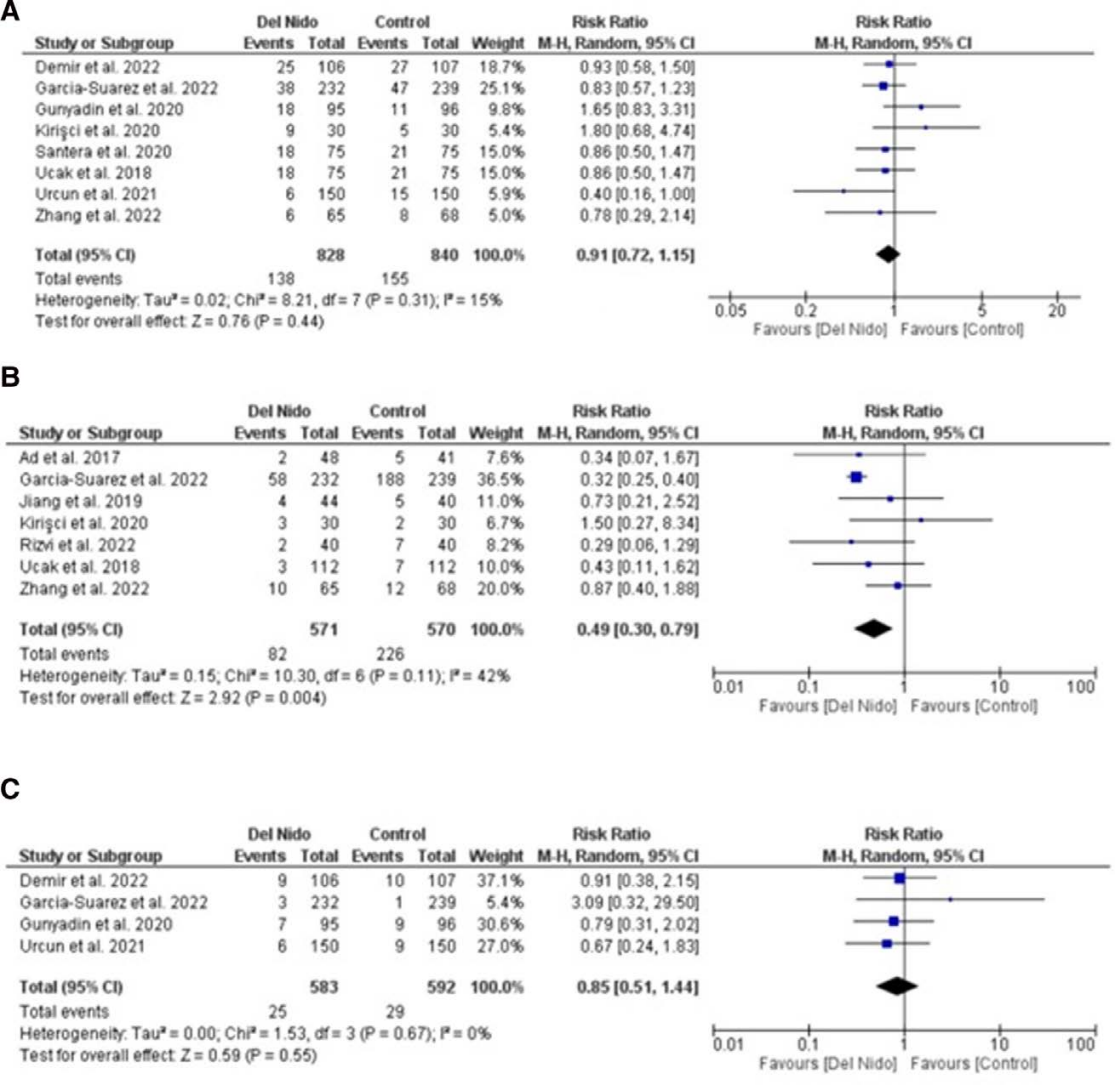
(A) Postoperative atrial fibrillation, (B) defibrillation after coronary reperfusion, and (C) intra-aortic balloon pump.

**Figure 5.**
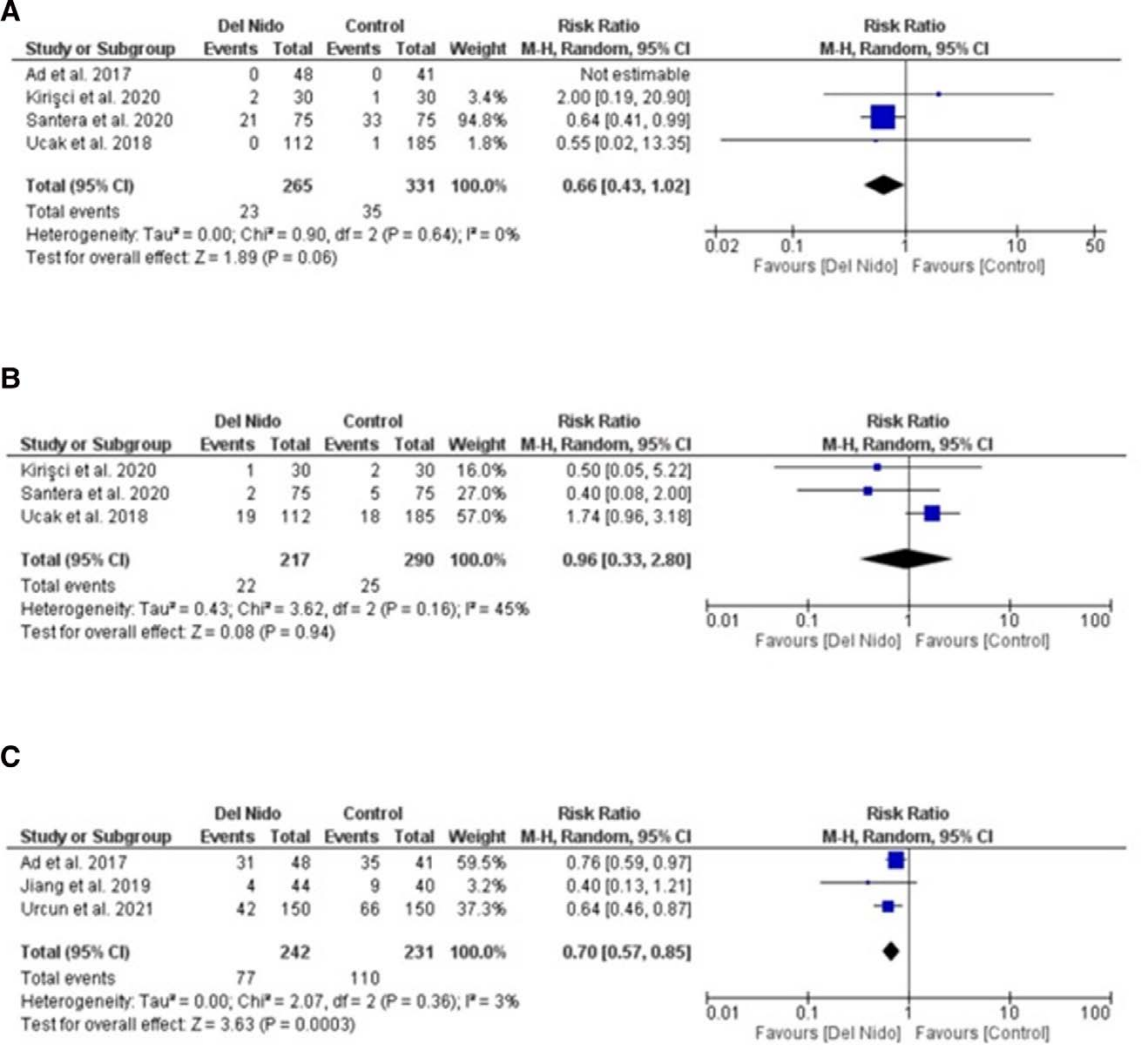
(A) postoperative kidney injury, (B) postoperative low cardiac output syndrome, and (C) inotropic support.

**Figure 6.**
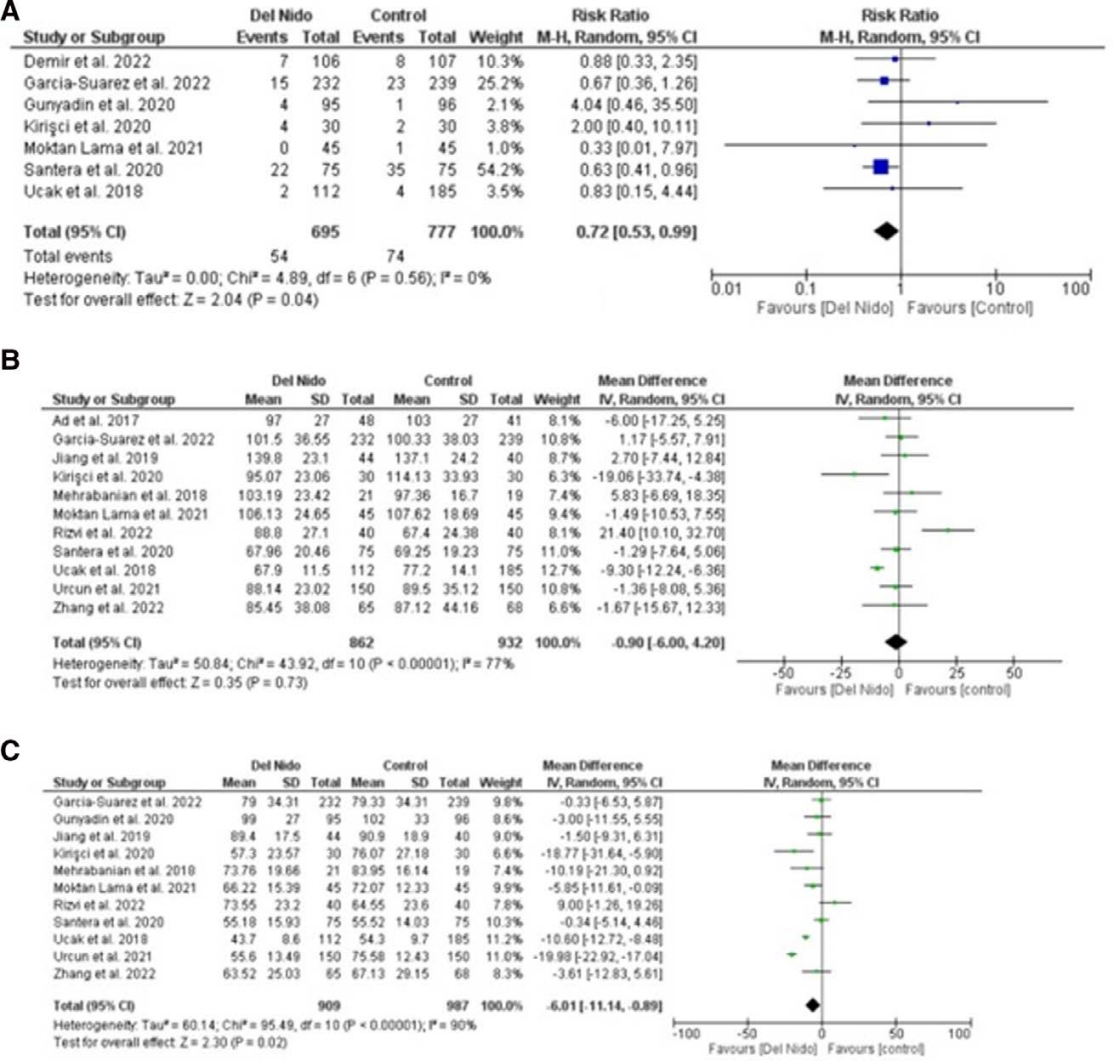
(A) Composite risk of stroke and/or TIA and/or kidney injury and mortality, (B) cardiopulmonary bypass time, and (C) cross-clamp time.

**Figure 7.**
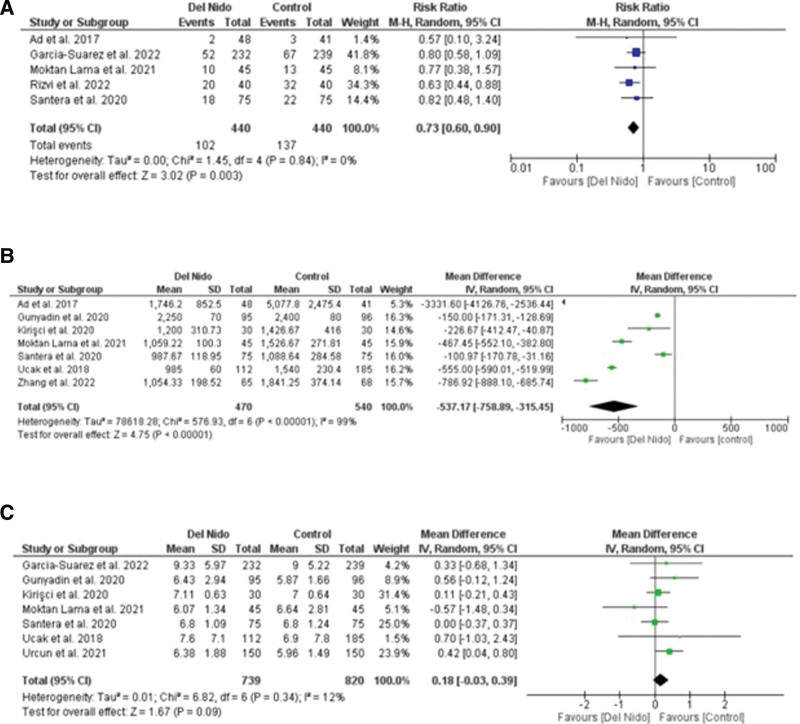
(A) Blood transfusion, (B) cardioplegia volume, and (C) hospital stay.

**Figure 8.**
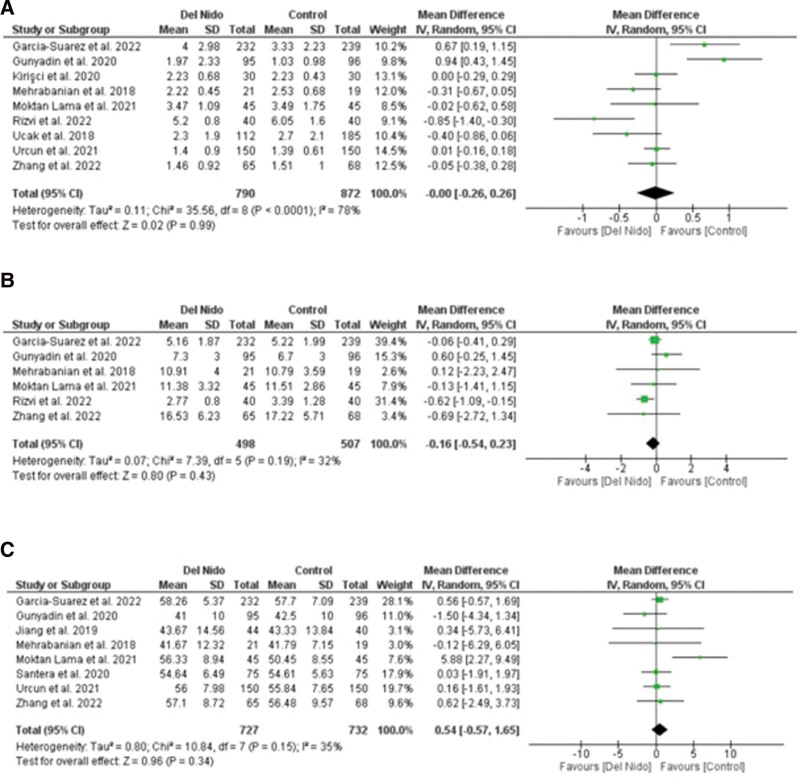
(A) Intensive care unit stay, (B) mechanical ventilation, and (C) postoperative left ventricular ejection fraction.

**Figure 9.**
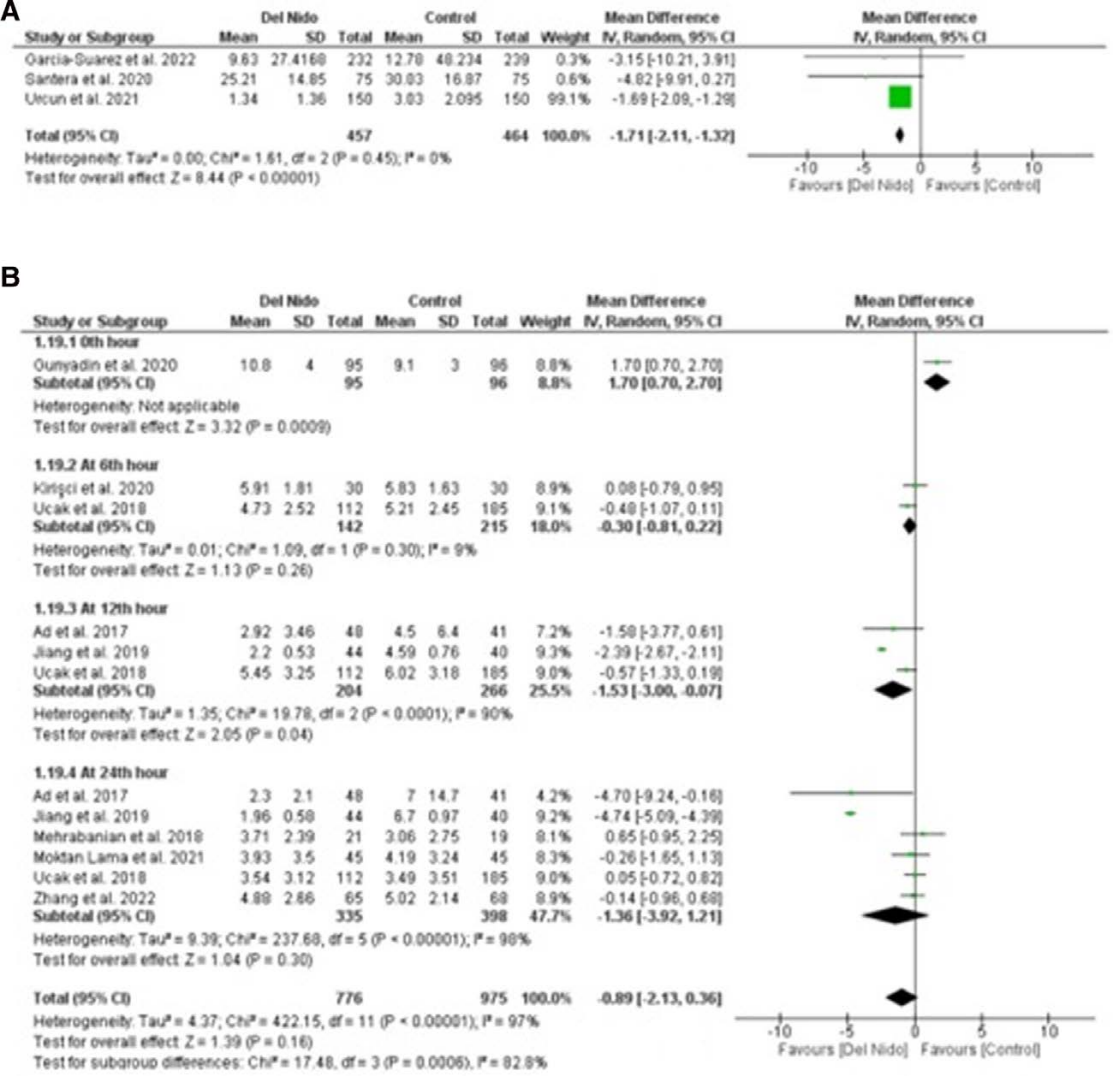
(A) Postoperative troponin T and (B) postoperative troponin I.

**Figure 10.**
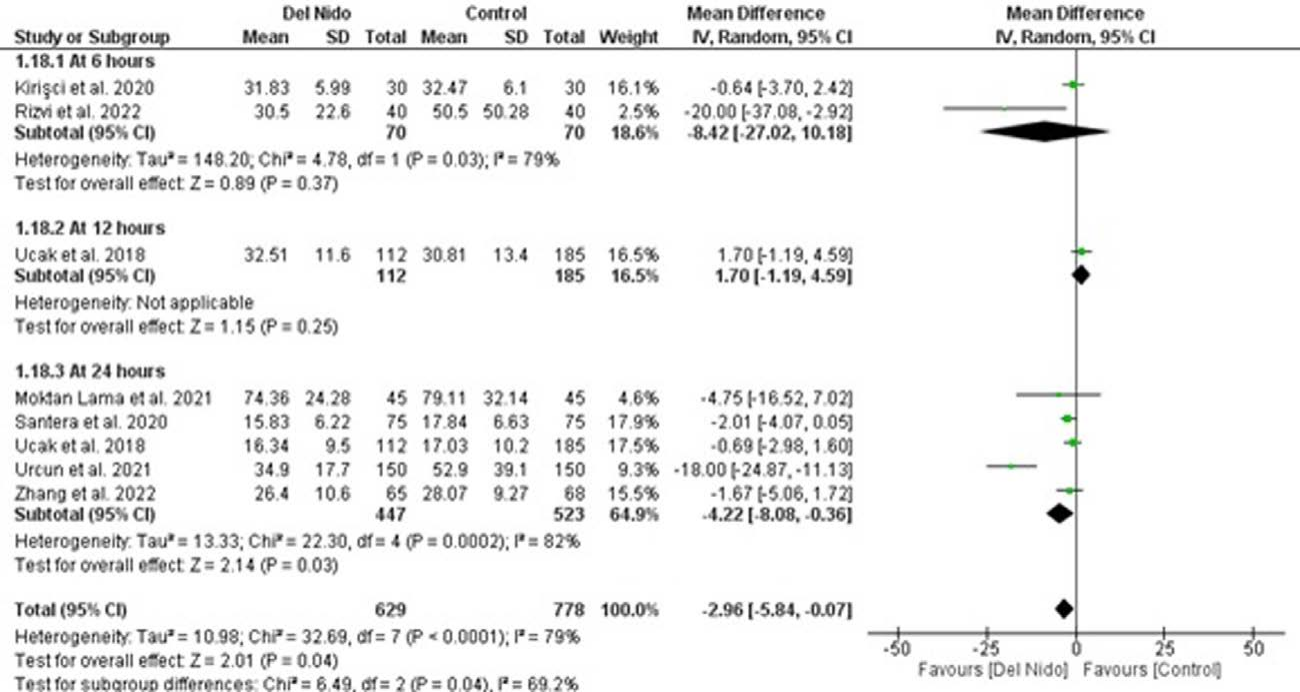
Postoperative CK-MB levels.

Five studies reported the outcome of spontaneous return of rhythm and significantly more patients in the Del Nido group returned to spontaneous rhythm as compared to the control group (RR: 1.58, 95% CI [1.02, 2.45]; *P* = .04; I^2^ = 94%) (Fig. 3A). Studies comparing postoperative MI showed no statistically significant difference between the 2 groups (RR:1.87, 95% CI [0.44, 7.99]; *P* = .40, I^2^ = 0%) (Fig. 3B). Similarly, no significant difference was seen in all-cause mortality (RR: 1.30, 95% CI [0.66, 2.56]; *P* = .44; I^2^ = 0%) (Fig. 3C), and POAF (RR: 0.91, 95% CI [0.72, 1.15]; *P* = .44; I^2^ = 15%) (Fig. 4A). Seven studies reported defibrillation after the cross-clamp was removed, and it shows that more patients in control group required defibrillation as compared to the Del Nido group. (RR: 0.49, 95% CI [0.30, 0.79]; *P* = .004; I^2^ = 42%) (Fig. 4.B). Additionally, IABP (RR: 0.85, 95% CI [0.51, 1.44]; *P* = .55; I^2^ = 0%) (Fig. 4C), postoperative kidney injury (RR: 0.66, 95% CI [0.43, 1.02]; *P* = .06; I^2^ = 0%) (Fig. 5A), and LCOS (RR: 0.96, 95% CI [0.33, 2.80]; *P* = .94; I^2^ = 45%) (Fig. 5B) were not significant. For inotropic support outcome, pooled result showed that patients in Del Nido group significantly required less inotropic support as compared to the control group (RR: 0.70, 95% CI [0.57, 0.85]; *P* = .0003; I^2^ = 3%) (Fig. 5C). Pooled result for composite risk of stroke and/or TIA and/or acute kidney injury and mortality showed significantly higher risk for control group as compared to the other group (RR: 0.72, 95% CI [0.53, 0.99]; *P* = .04, I^2^ = 0%) (Fig. 6A). From the continuous outcomes, studies reporting CPB time showed no significant association between the 2 groups (MD: −0.90, 95% CI [−6.00, 4.20]; *P* = .73; I^2^ = 77%) (Fig. 6B). However, cross-clamp time (MD: −6.01, 95% CI [−11.14, −0.89]; *P* = .02; I^2^ = 90%) (Fig. 6C), blood transfusion (RR: 0.73, 95% CI [0.60, 0.90]; *P* = .003; I^2^ = 0%) (Fig. 7A) and cardioplegia volume (MD: −537.17, 95% CI [−758.89, −315.45]; *P* < .00001; I^2^ = 99%) (Fig. 7B) were significantly increased in the control group. For the outcome of hospital stay (MD: 0.18, 95% CI [−0.03, 0.39]; *P* = .09; I^2^ = 12%) (Fig. 7C), ICU stay (MD: −0.00, 95% CI [−0.26, 0.26]; *P* = .99; I^2^ = 78%) (Fig. 8A), ventilation time (MD: −0.16, 95% CI [−0.54, 0.23]; *P* = .43; I^2^ = 32%) (Fig. 8B), and postoperative LVEF (MD: 0.54, 95% CI [−0.57, 1.65]; *P* = .34; I^2^ = 35%) (Fig. 8C) were found to be not significant. For cardiac markers, troponin T (MD: −1.71, 95% CI [−2.11, −1.32]; *P* < .00001; I^2^ = 0%) (Fig. 9A) and CK-MB (MD: −2.96, 95% CI [−5.84, −0.07]; *P* = .04, I^2^ = 79%) (Fig. 10) levels were significantly lower in Del Nido group as compared to the control group whereas troponin I (MD: −0.89, 95% CI [−2.13, 0.36]; *P* = .16, I^2^ = 97%) (Fig. 9B) did not show any significant difference between both the groups.

### 3.5. Assessment of heterogeneity

In order to enhance the reliability of the pooled estimate and mitigate potential biases, a sensitivity analysis was performed. This analysis entailed the exclusion of studies with either small or large sample sizes, as well as the removal of outliers. For the outcome of spontaneous rhythm return, Garcia-Suarez et al and Demir et al produced disproportionate effects and their exclusion significantly reduced heterogeneity from I^2^ = 94% to I^2^ = 41% (Figure S2, Supplemental Digital Content, http://links.lww.com/MD/N419). Similarly, in the outcome of CPB, removing the outliers Rizvi et al and Ucak et al, showed a significant decrease in heterogeneity from I^2^ = 77% to I^2^ = 8% while the pooled estimate remained not significant (Figure S3, Supplemental Digital Content, http://links.lww.com/MD/N419). In the outcome of cross-clamp time, Urcun et al and Ucak et al was removed resulting in decrease in heterogeneity and the pooled estimate was found to be not significant (MD: –2.97, 95% CI [–6.60, 0.67]; *P* = .11; I^2^ = 48%) (Figure S4, Supplemental Digital Content, http://links.lww.com/MD/N419). Gunyadin et al and Garcia-Suarez et al were removed from the ICU stay outcome which resulted in reduction of I^2^ value to 50% from 78% (Figure S5, Supplemental Digital Content, http://links.lww.com/MD/N419) For the outcome of troponin, I, removal of Jiang et al revealed reduction of heterogeneity to I^2^ = 66% (Figure S6, Supplemental Digital Content, http://links.lww.com/MD/N419). By excluding Urcun et al from the outcome of CK-MB levels, overall results were not significant with significant decrease in heterogeneity to (MD: –0.97, 95% CI [–2.59, 0.65]; *P* = .24; I^2^ = 38%) (Figure S7, Supplemental Digital Content, http://links.lww.com/MD/N419). Funnel plots for bypass time and cross-clamp time are exhibited in Figures S8 and S9, Supplemental Digital Content, http://links.lww.com/MD/N419.

### 3.6. Meta-regression

We assessed female sex and comorbidities such as diabetes mellitus and hypertension as a potential covariate affecting the effect size on our primary outcome, stroke and/or TIA. All the estimate of effects showed an insignificant association between each of these covariates and primary outcome. The results are as follows: female sex, Coeff: –0.0321, *P* = .4060; diabetes mellitus, Coeff: 0.0201, *P* = .2716; hypertension, Coeff: −0.0507, *P* = .3899 (Figures S10–S12, Supplemental Digital Content, http://links.lww.com/MD/N419).

## 4. Discussion

The results of this meta-analysis provide valuable insights into the effectiveness of DNC in adult cardiac surgery. The primary outcome analysis reveals that DNC has slightly lower incidence of postoperative stroke and/or TIA (2.44% in the DNC group vs 4.79% in the control) compared to other standard cardioplegia; however, this difference between the 2 groups could not reach statistical significance. A previous meta-analysis by Fresilli et al,^[16]^ which included 10 RCTs involving 1812 patients, also showed a similar trend in the incidence of postoperative stroke and/or TIA among the 2 groups (1.9% DNC vs 4.6% control), but their results were statistically significant. The results of our analysis differed due to inclusion of a study conducted by Demir et al^[22]^ which showed comparable incidence of postoperative stroke/TIA in the 2 groups. The lower incidence of stroke in the DNC group can be explained by the lower chance of micro-emboli formation owing to reduced need for re-dosing in DNC, as seen in this study by Mukdad et al^[23]^

The significantly higher proportion of patients with spontaneous rhythm return (65.53% for DNC vs 35.12% for other cardioplegias) associated with DNC suggests potential advantages in maintaining cardiac electrical stability during and after surgery. This finding is clinically relevant, as maintaining spontaneous rhythm can contribute to better postoperative outcomes and reduced arrhythmias. While analyzing the secondary outcomes, the authors found DNC to be more effective in terms of intraoperative outcomes: reduced need for inotropic support, decreased blood transfusion requirements, lower levels of troponin T and CK-MB, and a reduced cross-clamp time show that DNC led to relatively lower myocardial tissue damage. Furthermore, our results showed that significantly fewer patients required defibrillation after coronary reperfusion in the Del Nido group (14.36% in DNC vs 39.65%). This finding confirms that the DNC solution provides better myocardial protection compared to other conventional cardioplegia solutions used in adult cardiac surgery. Improved cardiac function intra and postoperatively also ensures adequate tissue perfusion to vital organs, leading to lower incidence of ischemic injuries. Moreover, the postoperative outcomes such as the incidence of atrial fibrillation and kidney injury also favored the Del Nido group due to the significantly decreased cross-clamp times (MD −6.01) observed in Del Nido group compared to the other cardioplegic solutions. The composite outcome of stroke, TIA, AKI, and mortality also showed a significantly lower risk in the Del Nido group as compared to control (7.77% DNC vs 9.52% control).

The Del Nido solution is a combination of a crystalloid solution (Plasma-LyteA) plus lidocaine, mannitol, magnesium sulfate, sodium bicarbonate, and potassium chloride.^[24]^ The re-dosage leads to accumulation of lidocaine in the cardiac tissue which presents as peripheral vasodilation, negative inotropism, ventricular arrhythmia, and seizures, among other side effects. Since re-dosing is only required in surgeries exceeding the 90-minute threshold for cross-clamp time, shorter cross-clamp times in the DNC group would mean lower need for re-dosage. The lower incidence of adverse events in the DNC group can also be attributed to significantly lower cardioplegia volumes required (MD: −537.17) and reduced need for re-dosage due to lower cross-clamp times in DNC compared to controls.

However, we did not find any improvement in terms of length of ICU and hospital stay in the Del Nido group compared to controls. Hence, we can conclude that the use of DNC improves intra and postoperative outcomes but does not affect the recovery time of patients undergoing cardiac surgery. On the contrary, our results show that Del Nido group had worse outcomes in terms of mortality (2.23% vs 1.69%) and MI (1.07% vs 0.56%), although these differences could not reach statistical significance.

Previous meta-analyses studying the efficacy and safety of DNC compared to other standard cardioplegia solutions in adult cardiac surgery have shown variable results. Li et al included 9 studies in their analysis and concluded that there was no difference in terms of myocardial enzymes, postoperative inotropic support, atrial fibrillation, hospital stay, and mortality between the Del Nido and conventional solutions.^[25]^ Additionally, a systematic review and meta-analysis by An et al pooled results from 1 RCT and 12 observational studies comparing DNC to blood cardioplegia. They concluded that patients in Del Nido group had a significantly lower cardioplegia volume requirement and lower aortic cross-clamp time. However, the Del Nido performed worse in terms of cardiac enzyme release.^[26]^ Another meta-analysis with a significantly large population of 21,779 owing to inclusion of 37 observational studies and 4 RCTs, found most outcomes to favor Del Nido group: decreased cardiac enzymes (troponin T and CK-MB), decreased CPB time, lower aortic cross-clamp time, lower cardioplegia volume requirement, and reduced need for intraoperative defibrillation.^[27]^

The results of this meta-analysis could have significant implications for clinical practice in adult cardiac surgery. The observed benefits of DNC, such as increased spontaneous rhythm return and reduced reliance on inotropic support, could translate into improved patient outcomes. Moreover, the potential reduction in postoperative complications like stroke and tissue damage suggests that DNC might enhance the overall safety and efficacy of cardiac surgeries. However, it is important to acknowledge that the clinical impact of these findings may vary depending on individual patient characteristics, procedural techniques, and surgical teams’ expertise. Clinicians should consider these findings alongside other relevant factors when making decisions about myocardial protection strategies in adult cardiac surgeries.

## 5. Limitations

While this meta-analysis provides valuable insights, it is important to recognize some limitations. A key limitation of our meta-analysis is the variability in cardioplegia solutions and surgical procedures across studies. Control groups employed diverse cardioplegias, affecting outcome interpretation. The inclusion of various surgeries added complexity. Subgroup analyses were limited by data availability and heterogeneous reporting, hindering robust analysis. Despite efforts to assess and address heterogeneity, some outcomes still displayed significant heterogeneity, which could affect the robustness of the findings. The included studies had moderate to high risk of bias, which could influence the reliability of the results. Future studies with rigorous methodology and larger sample sizes could provide more conclusive evidence. Although funnel plots were used to assess potential publication bias, it is important to acknowledge that publication bias can impact the validity of meta-analysis results. The findings may not be directly applicable to all patient populations, surgical techniques, or healthcare settings. Contextual factors should be considered when interpreting and applying the results. In the future, well-designed RCTs with larger sample sizes and more standardized methodologies could further validate the findings and provide a stronger foundation for clinical decision-making.

## 6. Conclusion

This meta-analysis presents a comprehensive evaluation of the benefits of DNC in adult cardiac surgery. The findings suggest potential advantages in terms of spontaneous rhythm return, reduced need for inotropic support, and decreased blood transfusion requirements. These findings hold promise for improving patient outcomes and warrant further exploration through well-designed clinical studies. However, it is important for clinicians to carefully consider the findings alongside individual patient characteristics and surgical context when determining the optimal myocardial protection strategy for adult cardiac surgeries.

## Author contributions

**Conceptualization:** Muhammad Ahmed.

**Data curation:** Muhammad Ahmed, Adarsh Raja, Shanta Bai, Johar Abbas.

**Formal analysis:** Adarsh Raja, Sandesh Raja.

**Investigation:** Vikash Virwani, Shanta Bai.

**Methodology:** Vikash Virwani, Sandesh Raja, Johar Abbas.

**Resources:** Syed Yawar Hussain.

**Software:** Syed Yawar Hussain.

**Supervision:** Mohammed Mahmmoud Fadelallah Eljack.

**Validation:** Abdul Moeed.

**Visualization:** Muhammad Sohaib Asghar.

**Writing – original draft:** Johar Abbas, Muhammad Sohaib Asghar.

**Writing – review & editing:** Abdul Moeed, Shanta Bai, Mohammed Mahmmoud Fadelallah Eljack, Muhammad Sohaib Asghar.

## Supplementary Material


