# Supplementary material for: Recent advances in Del Nido cardioplegia: A comprehensive analysis of randomized clinical trials in adult cardiac surgery
Source: Medicine (Baltimore). 2024 Sep 6;103(36):e39453. doi: 10.1097/MD.0000000000039453 (PMC11383727; doi:10.1097/MD.0000000000039453)
Supplement: Supplementary file 1 [file medi-103-e39453-s001.docx]

**Supplementary Table S1.** Detailed search strategy used in each database.

| PubMed | (Del Nido cardioplegia OR blood cardioplegia OR warm blood cardioplegia OR cold blood cardioplegia OR Bretschneider's solution OR St. Thomas cardioplegia OR Krebs Henseleit solution OR Custodial solution OR Plegisol solution OR St. Thomas Hospital Solution OR Buckberg cardioplegia OR multi-dose cardioplegia OR single-dose cardioplegia) AND (adult cardiac surgery OR acquired heart surgery OR open heart surgery OR myocardial protection OR Coronary artery bypass grafting OR CABG OR Valvular repair OR Valve surgery OR tricuspid repair OR TVR) |
| --- | --- |
| Scopus | (Del Nido cardioplegia OR blood cardioplegia OR warm blood cardioplegia OR cold blood cardioplegia OR Bretschneider's solution OR St. Thomas cardioplegia OR Krebs Henseleit solution OR Custodial solution OR Plegisol solution OR St. Thomas Hospital Solution OR Buckberg cardioplegia OR multi-dose cardioplegia OR single-dose cardioplegia) AND (adult cardiac surgery OR acquired heart surgery OR open heart surgery OR myocardial protection OR Coronary artery bypass grafting OR CABG OR Valvular repair OR Valve surgery OR "tricuspid repair OR TVR) |
| Cochrane Library | (Del Nido cardioplegia OR blood cardioplegia OR warm blood cardioplegia OR cold blood cardioplegia OR Bretschneider's solution OR St. Thomas cardioplegia OR Krebs Henseleit solution OR Custodial solution OR Plegisol solution OR St. Thomas Hospital Solution OR Buckberg cardioplegia OR multi-dose cardioplegia OR single-dose cardioplegia) AND (adult cardiac surgery OR acquired heart surgery OR open heart surgery OR myocardial protection OR Coronary artery bypass grafting OR CABG OR Valvular repair OR Valve surgery OR "tricuspid repair OR TVR) |

Supplementary Figure 1 (S1). Risk of bias summary.


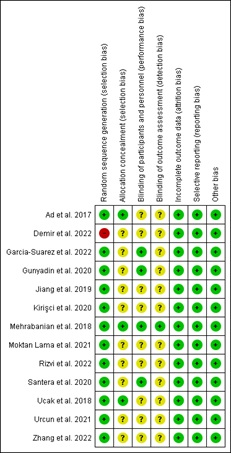


Supplementary Figure 2 (S2). Sensitivity analysis plot for spontaneous rhythm return.


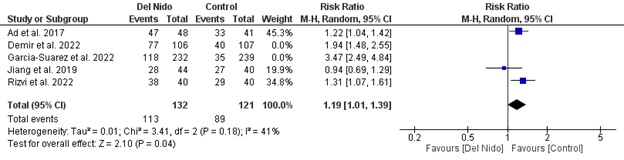


Supplementary Figure 3 (S3). Sensitivity analysis plot for cardiopulmonary bypass time.
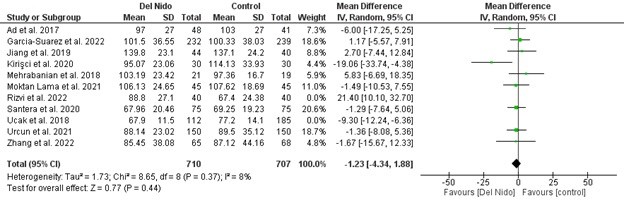


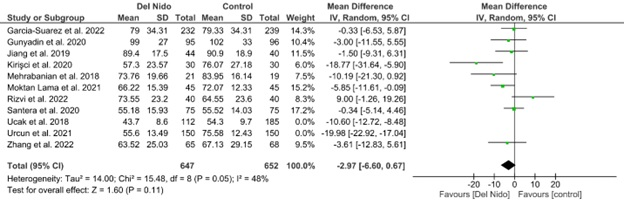
Supplementary Figure 4 (S4). Sensitivity analysis plot for cross-clamp time.


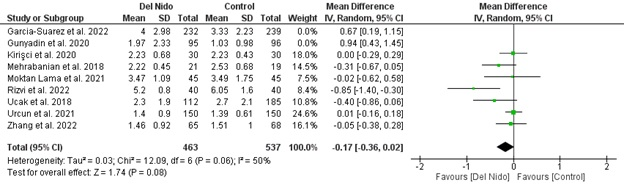
Supplementary Figure 5 (S5). Sensitivity analysis plot for ICU stay.


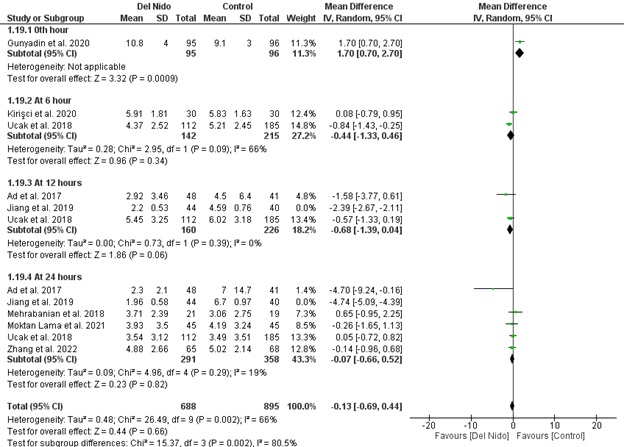
Supplementary Figure 6 (S6). Sensitivity analysis plot for troponin I level.


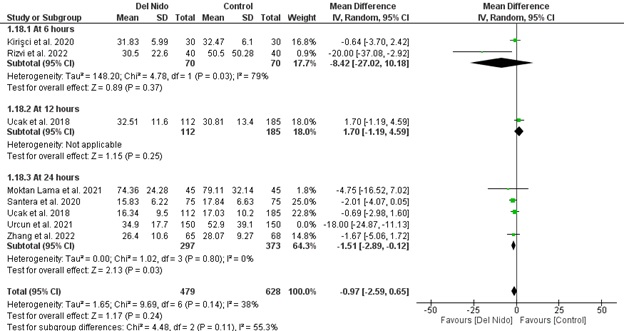
Supplementary Figure 7 (S7). Sensitivity analysis plot for CK-MB level.


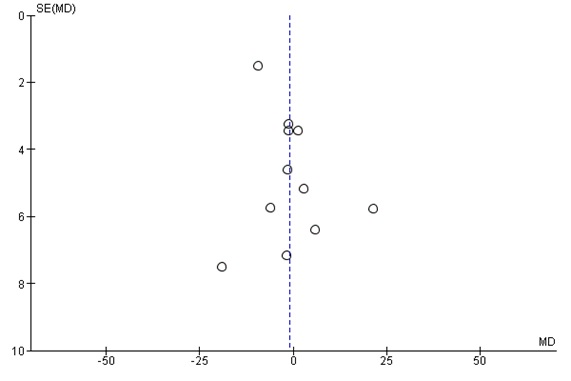
Supplementary Figure 8 (S8). Funnel plot for cardiopulmonary bypass time.


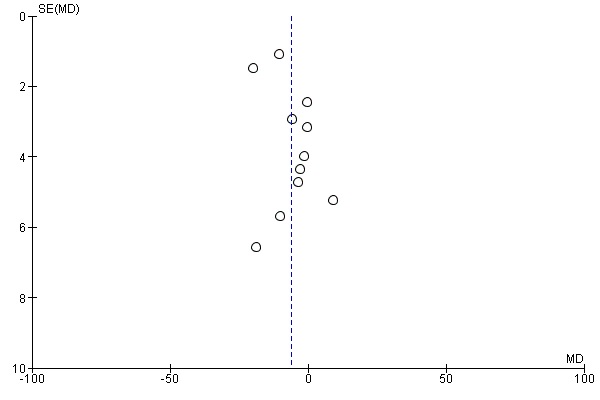
Supplementary Figure 9 (S9). Funnel plot for cross-clamp time.


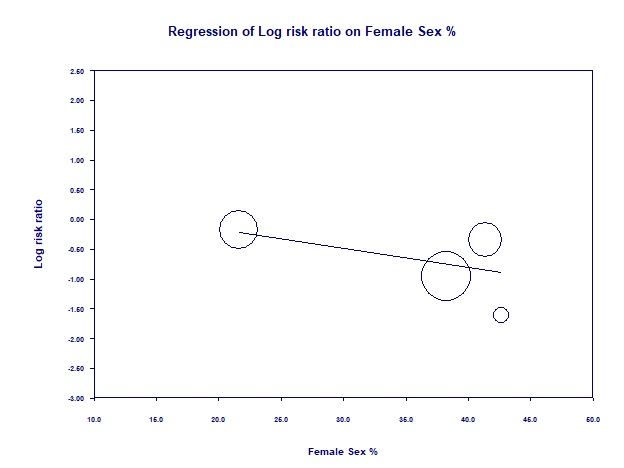
Supplementary Figure 10 (S10). Regression plot for Female sex %.


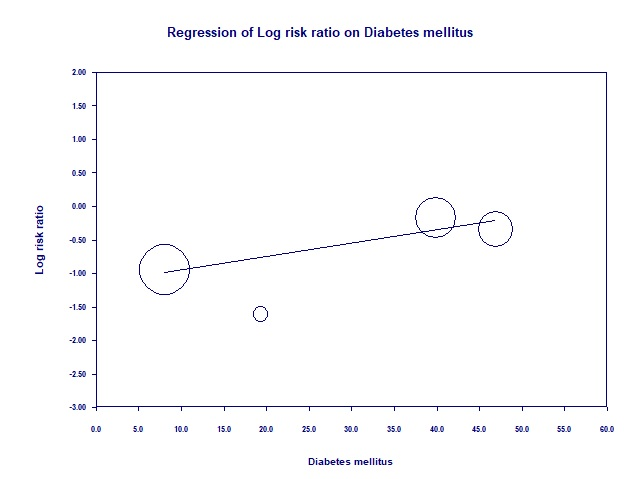
Supplementary Figure 11 (S11). Regression plot for Diabetes mellitus.


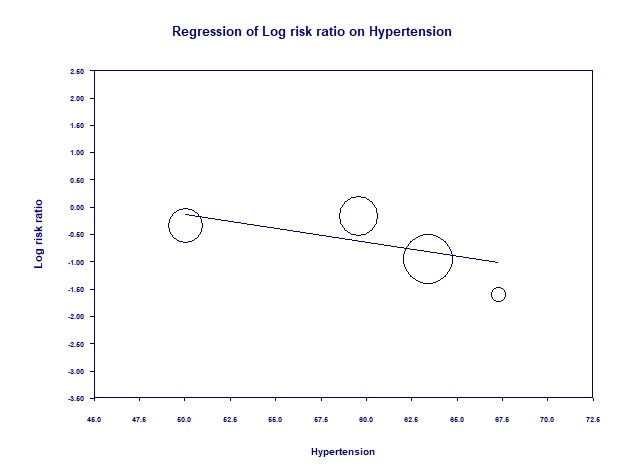
Supplementary Figure 12 (S12). Regression plot for Hypertension.
